# *Gemykibivirus* Genome in Lower Respiratory Tract of Elderly Woman With Unexplained Acute Respiratory Distress Syndrome

**DOI:** 10.1093/cid/ciz072

**Published:** 2019-02-02

**Authors:** Jian Wang, Yanpeng Li, Xi He, Jinmin Ma, Wenxin Hong, Fengyu Hu, Lingzhai Zhao, Qiongfang Li, Jianhui Zhang, Chiyu Zhang, Fuchun Zhang

**Affiliations:** 1 Institute of Infectious Disease, Guangzhou Eighth People’s Hospital, Guangzhou Medical University, Guangzhou; 2 The Joint Center for Infection and Immunity between Guangzhou Institute of Pediatrics, Guangzhou Women and Children’s Medical Center, and Institut Pasteur of Shanghai, Chinese Academy of Sciences (CAS), Shanghai; 3 Pathogen Discovery and Big Data Center, CAS Key Laboratory of Molecular Virology & Immunology, Institut Pasteur of Shanghai, Chinese Academy of Science, Shanghai; 4 BGI-Shenzhen, Shenzhen, China

**Keywords:** *Gemykibivirus*, acute respiratory distress syndrome, elderly woman, viral metagenomic analysis

## Abstract

Using metagenomics analysis, we are the first to identify the presence of a small, circular, single-stranded *Gemykibivirus* (GkV) genome from the respiratory tract of an elderly woman with severe acute respiratory distress syndrome. Our results suggest that further studies on whether GkVs infect humans and cause respiratory disease are needed.

*Gemykibivirus* (GkV) belongs to the *Genomoviridae* family, with a circular, single-stranded DNA (CRESS-DNA) genome that encodes a replication initiator protein (Rep) and capsid proteins (Cap) [1]. The *Genomoviridae* family contains 9 genera. More than 120 *Genomoviridae* genomes have been identified from various environmental samples (eg, air, sewage), plants, animals, insects, and humans by metagenomic sequencing [2]. GkV is the most commonly identified *Genomoviridae* virus in human samples by metagenomic sequencing, and has been found in the blood of a human immunodeficiency virus (HIV)–positive patient [3], brain and sera from multiple sclerosis patients [4], pericardial fluid of a patient with recurrent pericarditis [5], and cerebrospinal fluid of encephalitis patients [6, 7]. These studies reveal the widespread nature of the *Genomoviridae* and potential pathogenicity of GkVs in humans. Here, we report detection of a GkV genome in an elderly woman suffering unexplained acute respiratory distress syndrome.

## CASE REPORT

In January 2017, a 69-year-old woman presented with a low-grade fever accompanied by cough, chills, expectoration, and limb weakness. On the third day after symptom onset, she visited a local Red-Cross hospital in Guangzhou, China, because of worsening clinical conditions including shortness of breath and fever >39°C. Despite having received levofloxacin, cephalosporin, and oseltamivir for 2 days, her condition deteriorated further and she experienced hypoxemia on the fifth day after symptom onset. Chest radiograph showed infiltrates in the lower lung. The patient was transferred to Guangzhou Eighth People’s Hospital for further examination and treatment.

On hospital admission, she had a fever of 39.6°C, heart rate of 112 beats/min, respiratory rate of 22 breaths/min, and blood pressure of 134/78 mm Hg. She declared no prior immune suppression. Her detailed clinical characteristics are included in the Supplementary Materials. On physical exam, she was found to have extensive moist rales in the lung; on further testing, computed tomography showed bilateral diffuse infiltration in the lung (Supplementary Figure S1). The patient was diagnosed as having severe pneumonia and was treated with the following antiviral drugs: oseltamivir (150 mg twice daily), peramivir (600 mg daily), and an antibiotic drug moxifloxacin. On day 8, her body temperature returned to normal and clinical symptoms abated. The patient was discharged on day 12. She was found to be completely recovered on at a follow-up visit on day 29 and 6 months later (Supplementary Figure S1).

## EPIDEMIOLOGICAL AND LABORATORY INVESTIGATION

Epidemiological investigation found that the patient bought a live chicken 2 days before symptom onset and raised it at home. Her close contacts, husband and son, did not show any symptoms. The patient was initially suspected to have been infected with influenza viruses (H1N1 or H7N9). Samples for common respiratory pathogens, including 17 respiratory viruses, as well as mycoplasma and Chlamydophila pneumonia, were examined using a commercial multiplex real-time polymerase chain reaction (RT-PCR) assay (Guangzhou HuYanSuo Medical Technology Co., Ltd, China) and an in-house multiplex RT-PCR assay (Supplementary Methods). All samples were negative for these targeted pathogens.

Bacterial infections were tested and excluded by laboratory culture methods with selective medium; fungal infections were tested and excluded by 1,3-β-D glucan test and galactomannan test in the hospital clinical laboratories. These results suggested that the patient’s severe pneumonia was not caused by the above listed respiratory viruses or by common bacteria or fungi.

## METAGENOMIC SEQUENCING, WHOLE-GENOME SEQUENCING, AND PHYLOGENETIC ANALYSIS

**Figure 1.**
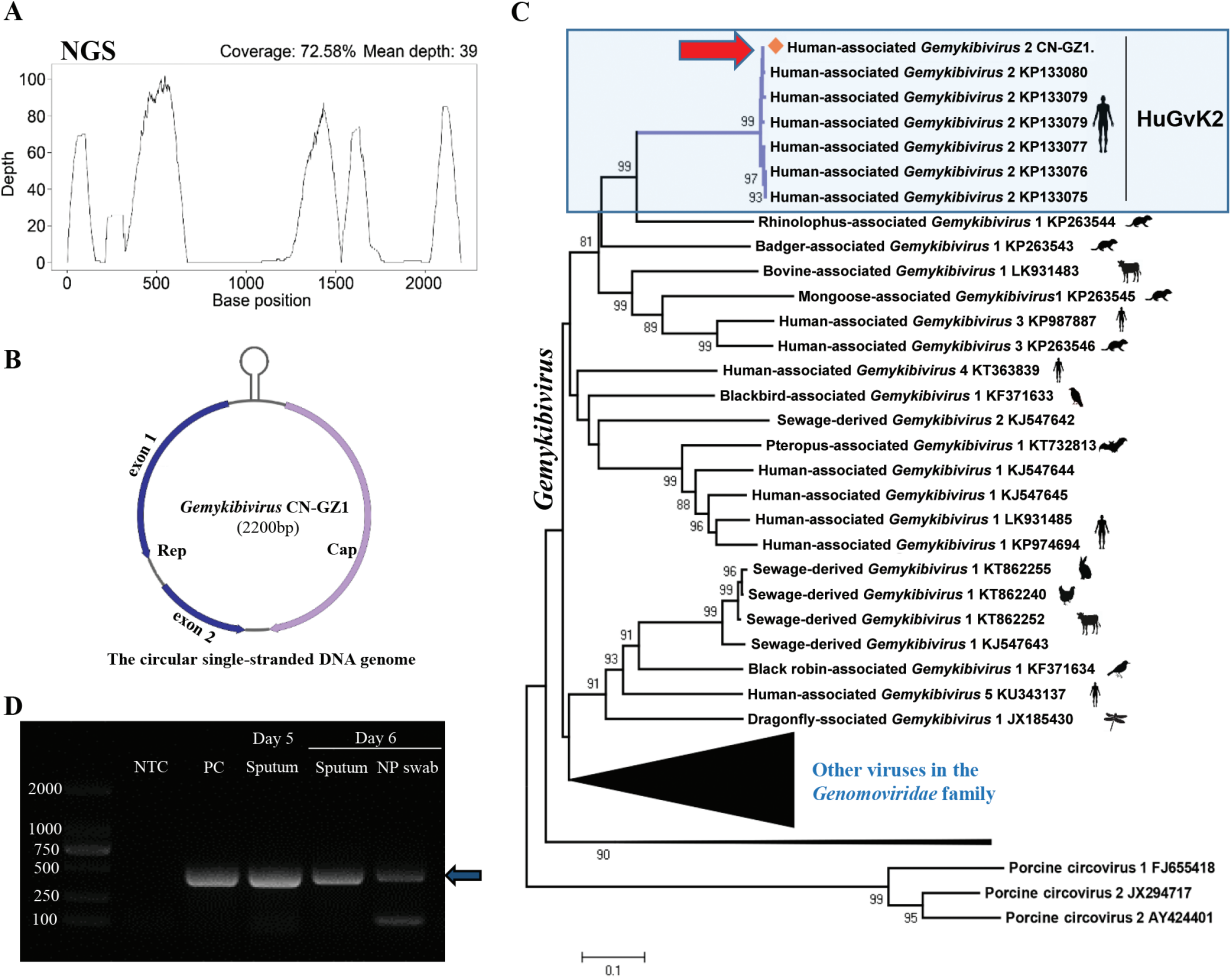
Genomic identification and detection of *Gemykibivirus* (GkV)_CN-GZ1. *A*, Genome coverage and sequencing depth by NGS. *B*, Genome organization. *C*, Neighbor-joining phylogenetic tree constructed by full-length genome of *Gemykibivirus* species and other viruses in the *Genomoviridae* family. GkV_CN-GZ1 is highlighted by an arrowhead. *D*, Detection of GkV_CN-GZ1 by a nested polymerase chain reaction assay. An arrowhead indicates the position of the specific products. All specific products from samples were confirmed by Sanger sequencing. Abbreviations: NGS, next generation sequencing; NTC: no template control. PC, positive control.

To discover the potential pathogen, we performed a viral metagenomic sequencing analysis using sputum collected on day 5 after symptom onset (Supplementary Methods). We found a high abundance of a GkV-related virus, which accounted for 72.7% of total viral reads. The mapped reads covered 72.6% of the reference GkV genome (GenBank: KP133075) (Figure 1A). The full-length genome of GkV (named GkV_CN-GZ1, GenBank: MH427642) was amplified from the original sample by a specific nested PCR and verified by Sanger sequencing (Supplementary Methods). The full-length genome that was obtained was 2200 nt in length (Figure 1B).

In order to determine the relationship between GkV_CN-GZ1 and existing GkV sequences, a phylogenetic tree was constructed using all the representative viral genomes from the *Genomoviridae* family (Figure 1C). The results showed that GkV_CN-GZ1 is closely clustered with several previously identified GkV strains (human-associated *Gemykibivirus* 2 (HuGkV2): BZ1/2 and SL1/2/3) from unexplained encephalitis and diarrhea patients [7] (Figure 1C). The GkV_CN-GZ1 genome shared 98.4% genetic similarity with the HuGkV2 strains [7]. Two deduced proteins (Rep and Cap) showed 99.7% and 100% similarity with the HuGkV2 strains. In particular, the GkV_CN-GZ1 genome, together with other HuGkV2 strains, was distinct from several other GkV strains that were also found in human samples, including patients with HIV, multiple sclerosis, recurrent pericarditis, and encephalitis [3–6].

## CONFIRMATION AND PREVALENCE SCREENING

To exclude the possibility of contamination from regents in our laboratory, we reextracted DNA from the sputum sample using another extraction kit (TIANamp Virus DNA/RNA Kit, TIANGEN, China) and tested the DNA sample using 2 PCR assays (Supplementary Methods). The results confirmed the presence of the GkV genome in the patient’s sample, but not in PCR control reagents. Further PCR tests excluded the possibility of contamination in transport medium used in sample collection. We then investigated the presence of GkV_CN-GZ1 in 30 patients with severe pneumonia from the same hospital using the specific PCR assay. All samples were negative for GkV_CN-GZ1.

To further exclude the possibility of laboratory contamination, we searched for any sequences similar to GkV_CN-GZ1 from the metagenomic sequencing data generated from more than 200 human swab samples (including another patient with severe pneumonia who was admitted to the same hospital during the same period [8]) with the same protocol and reagents in our laboratory. No GkV sequences were found. This indicated that the high abundance of GkV_CN-GZ1 in this patient was not likely to have been caused by external sources of contamination in the laboratory or hospital.

We then examined the presence of GkV_CN-GZ1 in the sputum and nasopharyngeal swab (NP) samples collected from the patient on day 6 using the nested PCR and probe-based RT-PCR (quantitative [q] PCR) methods (Supplementary Methods). Both samples had positive results by both PCR assays. In particular, sputum samples had brighter specific product bands than the NP samples (Figure 1D), indicating a higher viral concentration in the lower respiratory tract than upper respiratory tract. The sputum and NP specimens at day 6 had viral loads of 1.5×10^6^ and 1.2×10^3^ copies/mL, respectively (Supplementary Figure S2). The viral loads found in the sputum samples, as estimated by qPCR, were typical of those found with common respiratory virus infections (eg, 10^4.68^ to 10^8.52^ copies/mL for influenza viruses) [9].

In addition to GkV, other viral reads with low abundance (<5%) were found in the sputum. As nonpathogenic human viruses, the presence of *torque teno midi* virus 1 and 2 was unsurprising [10]. The presence of parvovirus, megavirus chiliensis, and murine leukemia virus was likely due to contamination by the nuclear acid extraction reagents as has been reported previously [11, 12]. Dragonfly-associated circular virus 1 and human herpesviruses 5 and 7 were excluded by specific PCR (possibly sequencing artifacts, as all the reads mapped to the same region of the reference sequence); other viruses (eg, cardiovirus, rabies virus) were also excluded due to very low abundance (<1%) or unmatched clinical symptoms.

## DISCUSSION

To date, no direct relationship has been established between GkV and any human diseases, although several viral strains were found among patients with encephalitis, diarrhea, pericarditis, and multiple sclerosis [3–7]. In the majority of the adult patients with pneumonia, a specific pathogen could not be found even when sensitive molecular diagnostic tools were used [13], rendering it difficult to design a therapeutic intervention. Here, we report for the first time the presence of GkV in a patient with severe pneumonia.

GkVs are divided into 3 large phylogenetic clades among which sequences identified from humans are dispersed (Figure 1C). The GkV_CN-GZ1 has genomic sequences that are almost identical to those of other HuGkV2 strains, and it shares high genomic similarity (>98%) with others that constitute the only lineage found in patient samples with various clinical symptoms in different countries including Sri Lanka, Brazil, and China [7]. This suggested a potential association between HuGkV2 and human diseases and raised the possibility that GkV may be an opportunistic pathogen.

Host tropism of most viruses in the *Genomoviridae* family are still unknown, with the exception of SsHADV-1, which has been shown to infect rice-associated fungi [2]. Because GkV_CN-GZ1 has low genetic similarity to SsHADV-1(50.6%), it is unlikely to show a similar tropism. Our finding of high viral loads from multiple specimens collected on different days from the upper and lower respiratory tract of this patient in the absence of an alternate pathogen implies a potential CN-GZ1 infection and associated pneumonia. Considering the widespread nature of the *Genomoviridae* family in the environment, it would be of interest to trace the actual host of this virus, and caution must be practiced with attempts to implicate these viruses in human infections and disease.

With the help of deep sequencing methods, more and more viruses from the *Genomoviridae* family will likely be identified. In those that present with disease, the potential pathologic effects of these viruses in humans warrant investigation. This is the first report of the GkV genome in a patient with severe pneumonia. Although the virus was not detected in 30 other severe pneumonia patients from the same hospital, the findings encourage a larger-scale investigation to elucidate the association between HuGkV2 and other GkV strains and human diseases.

## Supplementary Data

Supplementary materials are available at *Clinical Infectious Diseases* online. Consisting of data provided by the authors to benefit the reader, the posted materials are not copyedited and are the sole responsibility of the authors, so questions or comments should be addressed to the corresponding author.

ciz072_suppl_Supplementary_Materials

## Notes

***Acknowledgments.*** The authors thank Dr Feng Li from the Institute of Infectious Disease, Guangzhou Eighth People’s Hospital, for his kind suggestion on the project, and Prof Xia Jin at Institut Pasteur of Shanghai, Chinese Academy of Sciences (CAS), for English editing and critical reading of the manuscript.

***Financial support.*** This work was supported by grants from the National Science and Technology Major Project of China (2018ZX10101004003001, 2018ZX10102001 and 2017ZX10103009-002), the Strategic Priority Research Program (XDB29010000) and the “One Belt One Road” project (153831KYSB20170043) of the Chinese Academy of Sciences, the 133 project of Institut Pasteur of Shanghai, CAS, China Postdoctoral Science Foundation (2018M633025), and the Bureau of Science and Information Technology of Guangzhou Municipality (2014Y2-00550 and 201400000002).

***Potential conflicts of interest.*** All authors: No reported conflicts of interest. All authors have submitted the ICMJE Form for Disclosure of Potential Conflicts of Interest. Conflicts that the editors consider relevant to the content of the manuscript have been disclosed.
