# Supplementary material for: Gemykibivirus Genome in Lower Respiratory Tract of Elderly Woman With Unexplained Acute Respiratory Distress Syndrome
Source: Clin Infect Dis. 2019 Feb 2;69(5):861–4. doi: 10.1093/cid/ciz072 (PMC6695507; doi:10.1093/cid/ciz072)
Supplement: ciz072_suppl_Supplementary_Materials [file ciz072_suppl_supplementary_materials.docx]

**Supplementary Methods**

**Sample collection**

The investigation was approved by the Ethics Committee of Guangzhou Eighth People's Hospital and informed consent was obtained from the patient’s husband. Sputa were collected from the patient after atomization therapy with Sodium Chloride solution on Days 5 and 6 since symptom onset, and transferred into 3ml virus transport medium using swabs (Yocon Biology, Beijing, China). The nasopharyngeal swab (NP) was also collected on day 6. The samples were stored at -80 ℃ until use.

**Sample processing, nucleic acid extraction and random amplification**

A total of 200µl homogenized suspension from the Day 5 sputum sample was centrifuged for 15 min at 12,000g at room temperature, and filtered through a 0.45μm sterile filter (Corning, USA). Obtained suppernant was incubated with a cocktail of nucleases including 15U Turbo DNase (Invitrogen, USA), 20U Benzonase (Novagen, Germany) and 20U RNase I (Promega, USA) for 2hr, at 37°C. Total nucleic acids were extracted using QIAamp MinElute virus kit (Qiagen, Germay). A random-amplification approach (REPLI-g Single Cell WTA kit, Qiagen, Germany) was performed to obtain enough amplicons for library construction and metagenomic sequencing. The experiments above were performed at Institut Pasteur of Shanghai, Chinese Academy of Sciences.

**Library construction and next generation sequencing (NGS)**

The library construction and subsequent NGS were carried out according to the BGI-Seq500 sequencing protocol (National Gene Bank, BGI, China). Briefly, random amplified cDNA was purified by AmPure beads (Qiagen, Germany). The quantity and quality of purified cDNA were checked by Qubit Fluorometer 3.0 (Life Technologies, USA) and Agilent 2100 Bioanalyzer (Agilent Technologies Inc. USA). Then, a total amount of 1μg purified cDNA was fragmented to appropriate size (~250bp) by Covaris E210 (Covaris, USA), 3’-adenine overhangs were then added to the fragmented sequences. DNA fragments were ligated to BGI-Seq500 adapter, and the sequences were amplified for twelve cycles. Amplified products were purified again using AmPure beads (Qiagen, Germany), quantified with Qubit Fluorometer 3.0 (Life Technologies, USA), and the products were pooled to make a single strand DNA circle. DNA nanoballs (DNBs) were generated with the ssDNA circle by rolling circle amplification. The DNBs were loaded on the patterned arrays and sequenced on BGI-Seq500 platform (BGI, China). Single-end 100bp sequencing strategy was used.

**Bioinformatic analysis pipeline**

Raw data generated by NGS (BGI-Seq500 platform) was analyzed using an in-house method. Briefly, raw data was first filtered by SOAPnuke software to remove the sequencing adaptors, as well as low-quality and low-complexity reads. Sequences that belong to human host were then removed by mapping the reads to three human sequence databases (including a particular one of Chinese ethnicity) using SNAP software: HG19 (<http://hgdownload.soe.ucsc.edu/goldenPath/hg19/bigZips/hg19.2bit>), YH (<http://yh.genomics.org.cn/download.jsp>), refMrna (<http://hgdownload.soe.ucsc.edu/goldenPath/hg19/bigZips/refMrna.fa.gz>). The remaining reads were mapped using Kraken to a NCBI reference database with an E-value <10^-3^ for identification of viral sequences. Next, the above identified sequences were aligned (E-value <10^-3^) to the NCBI non-redundant nucleic acids database by BLAST. The reads that can mapped not only to viruses but also other species were discarded. In order to remove the false positive viral reads and achieve more accurate identification, all viral reads of each family were assembled into contigs by Minimo and IDBA-UD. The contigs and unassembled reads were blasted against the NCBI NT database. All identified viral sequences were kept and classified to the appropriate taxonomy levels.

**Full-length genome amplification and detection of GkV_CN-GZ1**

Nucleic acid was extracted from 200μl sputum and 100μl NP samples using QIAamp MinElute virus kit (Qiagen, Germay) and TIANamp Virus DNA/RNA Kit (TIANGEN, China), and eluted into 30μl RNase- and DNase-free water.

In order to obtain the full-length genome of GkV_CN-GZ1, we designed two sets of nested PCR primers based on the contig sequences of GkV_CN-GZ1 generated by NGS (Table 1). The PCR condition for nested PCR was 95°C 5min, 35 cycles for 95°C 30s, 50°C 30s, 72°C 1min, and final extension at 72°C for 10min.

To detect GkV_CN-GZ1 among clinical samples, we developed a real-time PCR (qPCR) assay. The primers and probe were listed in Table 1. The reaction were performed on a LightCycler 96 Real-Time PCR System (Roche Diagnostics, Germany) with amplification condition: 92 °C for 3 min, followed by 45 cycles of denaturation at 92 °C for 10s, annealing and extension at 55 °C for 30s. Threshold cycle (Ct) value was set to 0.05 automatically, and viral load of GkV_CN-GZ1was determined based on a standard curve.

**Table 1. The primer information.**

| **Assay** | **Primer set** | **Primer name** | **Sequence (5’-3’)** |
| --- | --- | --- | --- |
| Nested-PCR | Primer set 1 | Outer-F1  Outer-R1  Inner-F1  Inner-R1 | GCGTCATTTTCGAGGGAACG  ATAGGTCGTGGGAGGAGAGG  ATGCCAAACCGGATAAGAGG  TTTCGGCGGCTTGTTTTCAC |
|  | Primer set 2 | Outer-F2  Outer-R2  Inner-F2  Inner-R2 | ATACGGCTCTGGATCTTCGC  CTAGCACGTGAACGGGAGAA  TGGGCAAAGCAGGTACATAGA  CGGTAGCGAGAACCATAAGC |
| qPCR |  | Gem-F  Gem-R  Gem-Probe | GGGCAAAGCAGGTACATAGAG  CGATCAACTGGATGGCATGATA  (FAM)AACTCCTCTCGACTTGTTGCAGCG(BHQ1) |

**Detection of common respiratory pathogens using multiplex RT-qPCR assays**

Common respiratory pathogens causing pneumonia, including viruses and bacteria were tested for the sputum sample at Day 5 of symptom onset by a commercial multiplex RT-qPCR kit (Guangzhou HuYanSuo Medical Technology Co., Ltd, Guangzhou, China). The panel covered 17 different respiratory pathogens, including influenza (IFV) A and B viruses, rhinovirus, bocavirus, adenovirus, enterovirus, parainfluenza virus (PIV1-4), respiratory syncytial virus (RSV), human metapneumovirus (HMPV), coronavirus (HCoV-229E, OC43, NL63 and HKU1), mycoplasma pneumoniae (MP), and chlamydophila pneumonia. All samples including sputum and NP samples were further tested using our in-house multiplex RT-qPCR assay that covers 17 common respiratory viruses (IFV-A, IFV-B, IFV-C, RSV, HMPV, PIV1–4, enterovirus, rhinovirus, CoV-229E, CoV-OC43, CoV-NL63, CoV-HKU1, adenovirus and bocavirus).

**Supplementary Results**

**Clinical characteristics of the patient on admission (Day 5 of symptom onset)**

Laboratory examination showed the patient had normal white blood cell count of 3.3×10^9^/L(normal, 3.5-9.5×10^9^/L), decreased lymphocyte count of 0.37×10^9^/L (normal, 1.1-3.2×10^9^/L), reduced platelet count (PLT) of 77×10^9^/L (normal, 100-350×10^9^/L), elevated C-reactive protein level at 62.2 mg/L (normal, <8.0 mg/L), normal hemoglobin (HB) level at 115 g/L (normal, 110-150g/L), decreased oxigenation Index (OI) of 339 mmHg (normal, 400-500mmHg)，reduced CD3 T cell count of 318/mm^3^ (normal, 500-1500/mm^3^), reduced CD4 T cell count of 209/mm^3^ (normal, 300-1200/mm^3^), and reduced CD8 T cell count of 80/mm^3^ (normal, 238-874/mm^3^).


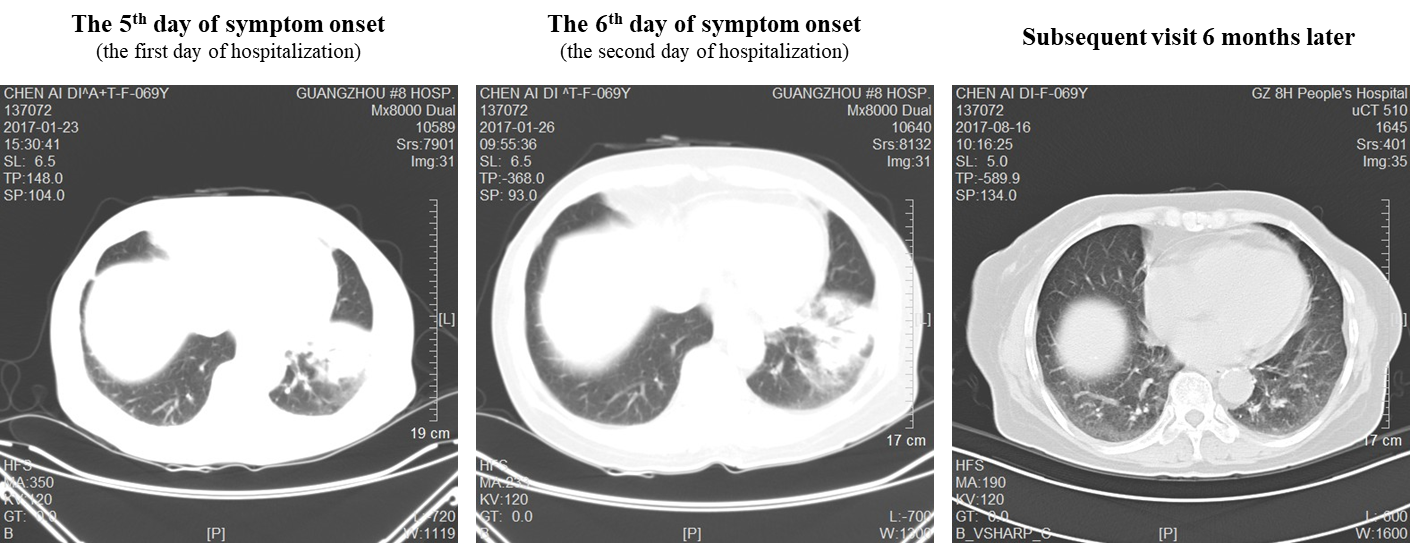


**Supplementary Figure 1. CT images of patient’s lung.**

**
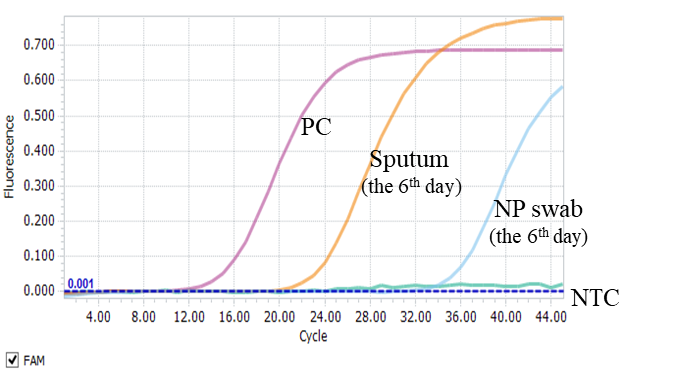
**

**Supplementary Figure 2. Amplification curves of the sputum and NP samples collected at Day 6.** PC, positive control. NTC, non-template control.
